# Successful Use of Impella Support to Treat Cardiogenic Shock Secondary to Takotsubo Cardiomyopathy Owing to Alcohol Withdrawal

**DOI:** 10.7759/cureus.89953

**Published:** 2025-08-12

**Authors:** Yuki Takeuchi, Tokutada Sato

**Affiliations:** 1 Intensive Care Unit, Showa Medical University Fujigaoka Hospital, Yokohama, JPN

**Keywords:** alcohol withdrawal, cardiogenic shock, convulsion, impella, takotsubo cardiomyopathy

## Abstract

Takotsubo cardiomyopathy associated with alcohol withdrawal is rare in Japan, and its management in such cases using percutaneous left ventricular assist devices (Impella; Abiomed, Japan) is not common. This report describes the treatment of a patient with cardiogenic shock resulting from alcohol withdrawal-induced Takotsubo cardiomyopathy using Impella support. A female patient in her 60s with a history of alcohol dependence presented to the emergency room with fever and convulsions. Upon arrival, she developed status epilepticus requiring intubation and mechanical ventilation. Subsequently, her blood pressure decreased, leading to shock. On day two of hospitalization, laboratory tests revealed elevated creatine kinase and troponin I levels, and an electrocardiogram demonstrated ST elevation. Transthoracic echocardiography demonstrated a reduced left ventricular ejection fraction of approximately 30-40%. Coronary angiography revealed no significant stenoses. Left ventricular angiography indicated akinesis in the mid-ventricle, with hyperkinesis at the base and apex. Based on the Mayo Clinic criteria, the patient was diagnosed with mid-ventricular Takotsubo cardiomyopathy. Myocardial biopsy ruled out myocarditis. Furthermore, CT revealed no obvious adrenal tumor, and pheochromocytoma was also ruled out. No evidence of alternative causes such as emotional stressors, severe infection, or exogenous catecholamine administration was found; thus, alcohol withdrawal was identified as the likely underlying trigger. The hemodynamic data showed a left ventricular pressure of 99/10 mmHg, an aortic pressure of 79/58 mmHg, with a significant intraventricular pressure gradient, and an elevated left ventricular end-diastolic pressure (LVEDP) of 33 mmHg. Right heart catheterization performed simultaneously revealed a pulmonary artery pressure of 46/31/37 mmHg, a pulmonary capillary wedge pressure of 32/30/29 mmHg, a cardiac output (CO) of 3.16 L/minute, and a cardiac index of 2.16 L/minute/m². The patient was in cardiogenic shock and required mechanical circulatory support. Given the presence of an elevated intraventricular pressure gradient and LVEDP, we judged that the Impella device would provide adequate circulatory support while unloading the left ventricle to reduce LVEDP and wall stress without exacerbating the left ventricular outflow tract gradient. An Impella CP was inserted and initiated at a support level of P8. Concurrently, antiepileptic therapy was administered for status epilepticus. On hospital day three, the LVEDP decreased while CO and cardiac power output (CPO) improved, indicating effective left ventricular support. While targeting a mean arterial pressure >65 mmHg, CPO >0.6 W, pulmonary artery pulsatility index >0.9, and lactate <2.0 mmol/L as reference parameters, the support level was gradually reduced. On hospital day five, CO recovered to 5.0 L/minute, and the Impella support level was successfully weaned to P3, allowing for device removal. The patient was discharged from the intensive care unit on hospital day 12. This case suggests that Impella support may provide appropriate hemodynamic stabilization in Takotsubo cardiomyopathy complicated by cardiogenic shock. However, as this is a single case report, further accumulation of similar cases is warranted in the future.

## Introduction

Takotsubo cardiomyopathy is a transient myocardial disorder triggered by emotional or physical stress, characterized by reversible left ventricular dysfunction [1]. The prevalence of Takotsubo cardiomyopathy among all hospitalizations is approximately 0.02% [2]. Among patients presenting with suspected acute coronary syndrome who undergo coronary angiography, Takotsubo cardiomyopathy accounts for 2.1% of cases [3]. Although an association between alcohol withdrawal and Takotsubo cardiomyopathy has also been reported, only a small number of case reports exist, indicating that this condition is rare. Alcohol is considered a central nervous system depressant. The inhibitory tone is enhanced by modulating the activity of the gamma-aminobutyric acid, whereas the excitatory tone is suppressed by modulating the activity of the excitatory amino acid such as glutamate. Adaptation occurs by increasing the number of glutamate receptors to maintain a normal state of excitation. This homeostasis is maintained in the presence of ethanol. A sudden interruption of alcohol intake disrupts this balance, leading to hyperactivity of the central nervous system and uncontrolled neuronal overactivity. Hyperactivation of the sympathetic nervous system, leading to excessive catecholamine release, has been implicated as a potential trigger for Takotsubo cardiomyopathy [4,5]. However, Takotsubo cardiomyopathy is generally triggered by psychological or physical stressors, such as medication, infection, or stroke; therefore, it is necessary to investigate potential causes other than alcohol withdrawal [1]. The diagnostic criteria are often based on the original Mayo Clinic criteria, which require the exclusion of cardiomyopathies and pheochromocytoma [6].

Takotsubo cardiomyopathy can be classified into several anatomical variants based on the pattern of regional wall motion abnormalities. The typical type is the apical type, which accounts for the majority of cases. Atypical types include the mid-ventricular type, basal type, and focal type [1]. The mid-ventricular type is observed in approximately 10-20% of cases and is more prone to causing severe acute left heart failure [7]. In addition, approximately 19-25% of patients with Takotsubo cardiomyopathy develop left ventricular outflow tract (LVOT) obstruction, which can lead to an increased left ventricular-aortic pressure gradient and elevated left ventricular end-diastolic pressure (LVEDP) [8,9]. If a patient with LVOT obstruction develops cardiogenic shock, mechanical circulatory support should be considered. In such cases, intra-aortic balloon pumping (IABP) may worsen the pressure gradient caused by LVOT obstruction. The Impella device can maintain sufficient circulatory flow and reduce LVEDP by unloading the left ventricle without exacerbating the left ventricular-aortic pressure gradient, thereby reducing left ventricular wall stress and promoting recovery of cardiac function [10]. In contrast, in cases of severe shock, circulatory support with extracorporeal membrane oxygenation (ECMO) may be required. According to previous reviews, the proportion of mechanical circulatory support used for Takotsubo cardiomyopathy complicated by cardiogenic shock was ECMO at 50%, Impella at 36%, IABP at 10%, and others at 4% [11].

## Case presentation

A female in her 60s (height: 162 cm; weight: 37 kg) had no notable medical history. Her regular medications included acamprosate (999 mg, thrice daily) and mecobalamin (1.5 mg, thrice daily). The patient had been diagnosed with alcohol dependence 10 years prior (year Y-10) and was undergoing treatment. However, the patient continued to consume alcohol. In mid-March of year Y, she developed a fever and sore throat, but continued to drink heavily at night. After her family intervened to stop her alcohol consumption on March X-2, she experienced tonic seizures and was transported to our emergency department by ambulance on March X. On admission, vital signs were as follows: blood pressure, 109/80 mmHg; heart rate, 115 beats/minute; respiratory rate, 32/minute; SpO₂, 99% on 10 L/minute oxygen via a reservoir mask; and body temperature, 39.1°C. Her level of consciousness was E1V1M3, according to the Glasgow Coma Scale. Auscultation revealed coarse crackles in both the anterior lung fields. Heart sounds were regular, with no murmurs. She continued to exhibit tonic seizures upon arrival, demonstrating difficulty in making an obedience motion; however, no obvious motor deficits in the limbs were observed. Arterial blood gas analysis under 10 L/minute oxygen via a reservoir mask showed a pH of 6.926, PaO₂ of 100 mmHg, PaCO₂ of 91.8 mmHg, HCO₃⁻ of 20.5 mmol/L, and lactate level of 16.19 mmol/L, suggesting type II respiratory failure and mixed acidosis with lactic acidosis (Table 1). Blood test results revealed elevated levels of white blood cells, aspartate aminotransferase, alanine aminotransferase, creatine kinase (CK), troponin I, and ammonia (Table 1).

**Table 1:** Laboratory results on admission. pH: power of hydrogen; PaO₂: partial pressure of arterial oxygen; PaCO₂: partial pressure of carbon dioxide; HCO₃⁻: hydrogen carbonate

Laboratory parameter	Value	Reference values	Units
pH	6.926	7.35–7.45	mmHg
PaO_2_	100	80–100	mmHg
PaCO₂	91.8	35–45	mmol/L
HCO_3_^-^	20.5	23–28	mmol/L
Lactate	16.19	<1.3	mmol/L
White blood cells	13.2	3.3–8.6	×10^3^/μ
Hemoglobin	11.8	11.6–14.8	g/dL
Platelets	112	158–348	×10^3^/μL
Aspartate aminotransferase	170	13–30	IU/L
Alanine aminotransferase	80	7–42	IU/L
Creatine kinase	2437	41–151	IU/L
Creatine kinase-myocardial band	20.7	≦3.1	ng/mL
Troponin I	1506	≦26.2	pg/mL
Ammonia	366	15–49	μg/mL
C-reactive protein	0.19	0.00–0.14	mg/dL
Blood glucose	196	73–109	mg/dL
Procalcitonin	0.03	<0.05	ng/mL
Blood culture	Negative	-	-

**Figure 1:**
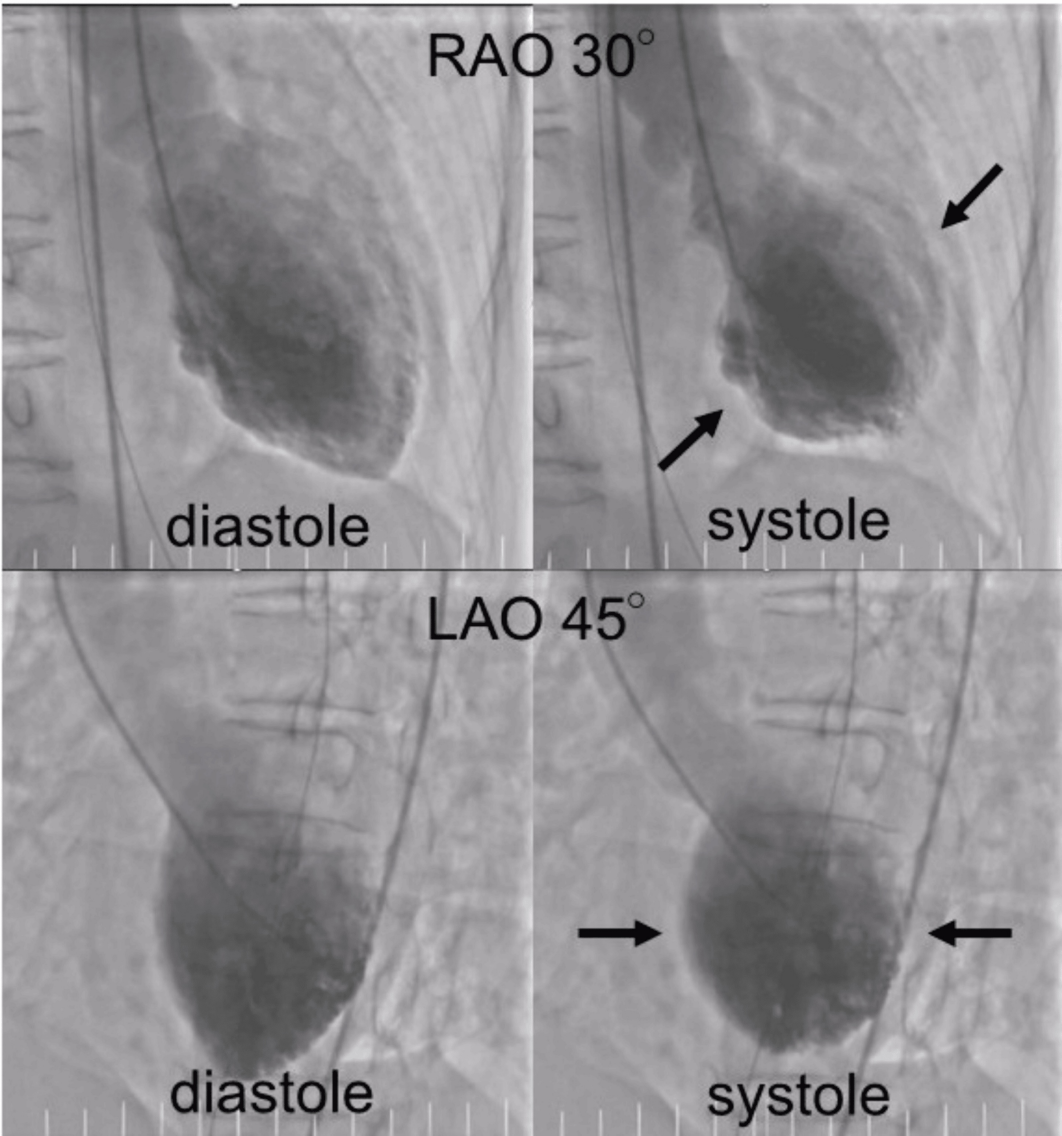
Left ventriculography. Segment 2/4/6/7 akinesis (→), segment 1/3/5 hyperkinesis, left ventricular pressure 99/10/33 mmHg, aortic pressure 79/58/67 mmHg.

Hypoglycemia was ruled out. Urinalysis showed no pyuria or ketonuria. ECG revealed ST-segment elevation in leads V3-V5. Cranial CT showed no space-occupying lesions; however, chest CT demonstrated pulmonary congestion and shadows, suggestive of pneumonia. Based on clinical presentation, acute symptomatic seizures due to alcohol withdrawal and status epilepticus were diagnosed. Tonic seizures persisted after arrival, lasting approximately 15 minutes, and the patient was intubated and treated with midazolam and levetiracetam. After the initiation of mechanical ventilation, her blood pressure began declining. Although the sedative dosage was adjusted considering its side effects, there was no improvement. Therefore, septic shock secondary to pneumonia was suspected, and aggressive fluid resuscitation with norepinephrine, antibiotics, and corticosteroids was initiated. Thiamine supplementation was initiated promptly because of a suspected deficiency in chronic alcohol use, although pre-treatment levels were not measured. Although transthoracic echocardiography demonstrated a reduced left ventricular ejection fraction of approximately 30-40%, no clear regional wall motion abnormalities were observed. Considering that the initial symptom was not chest pain but seizures, and that CK-MB elevation was minimal compared to total CK, we suspected myocardial injury associated with status epilepticus or septic shock rather than a primary acute myocardial infarction. However, the shock persisted on hospital day two despite fluid resuscitation and increased norepinephrine levels. Follow-up blood tests showed further elevation of cardiac markers (CK, 4,277 IU/L; CK-MB, 37.5 ng/mL; and troponin I, 21,331 pg/mL), and ECG revealed ST elevation in leads V2-V6 with T-wave flattening. Coronary angiography revealed no significant stenosis, whereas left ventriculography showed akinesis of the mid-ventricular segments and hyperkinesis of the basal and apical regions, with the regional wall motion abnormalities extending beyond a single coronary artery territory (Figure 1).

**Figure 2:**
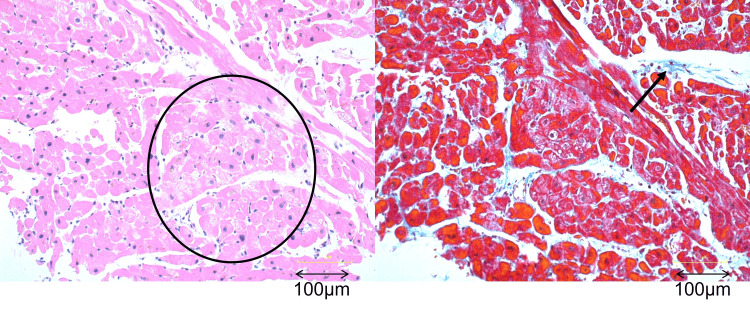
Pathological findings of myocardial biopsy (hematoxylin and eosin stain, 40× and Masson trichrome stain, 40×). Some myocardial cells exhibit degeneration (〇); however, the interstitial fibrosis is mild (→), and there is no infiltration of inflammatory cells. These findings suggest some form of cardiomyopathy, but are nonspecific.

Hemodynamic data showed a left ventricular pressure of 99/10 mmHg, aortic pressure of 79/58 mmHg, and an elevated LVEDP of 33 mmHg. Right heart catheterization revealed a pulmonary artery pressure of 46/31/37 mmHg, pulmonary capillary wedge pressure of 32/30/29 mmHg, cardiac output (CO) of 3.16 L/minute, and cardiac index of 2.16 L/minute/m². Given the differential diagnosis of Takotsubo cardiomyopathy and myocarditis, a myocardial biopsy was performed. Histopathological examination revealed mild degeneration in a limited number of cardiomyocytes without inflammatory cell infiltration, suggestive of nonspecific cardiomyopathy (Figure 2).

Myocarditis was ruled out based on the pathological findings, and no adrenal tumor was detected on CT, making pheochromocytoma unlikely. Therefore, in accordance with the Mayo Clinic criteria, the patient was diagnosed with mid-ventricular-type Takotsubo cardiomyopathy [6]. Furthermore, severe infection and catecholamine-induced drug effects were considered unlikely as causes, leading to the conclusion that the Takotsubo cardiomyopathy was triggered by alcohol withdrawal. Given the presence of cardiogenic shock, mechanical circulatory support was initiated using an Impella CP device at support level P8. During Impella support, continuous intravenous infusion of unfractionated heparin was administered for anticoagulation, with the dose adjusted to maintain an activated clotting time target of approximately 180 seconds. This led to rapid hemodynamic stabilization and shock resolution, and norepinephrine was promptly tapered. The support level was gradually reduced while targeting a mean arterial pressure (MAP) >65 mmHg, cardiac power output (CPO) >0.6 W, pulmonary artery pulsatility index (PAPi) >0.9, and lactate <2.0 mmol/L. By hospital day three, the LVEDP had decreased to 12 mmHg, the blood pressure had increased to 101/78 mmHg, the CO had risen to 3.7 L/minute, and lactate levels had declined, indicating adequate left ventricular support. In addition, CK-MB had decreased to 18.2 ng/mL and troponin I to 17,402 pg/mL, suggesting that the myocardial injury caused by Takotsubo cardiomyopathy had passed its peak. On hospital day four, the PAPi, suggesting right ventricular function, had improved from 0.8 to 2.4. By hospital day five, the CO had improved to 5.0 L/minute, and the support level was reduced to P3, allowing for Impella removal (Table 2).

**Table 2:** Hemodynamic data. On hospital day two, an Impella CP was inserted. On hospital day three, LVEDP is decreased, while CO and CPO increased, indicating effective left ventricular support. PAPi, a marker of right ventricular function, also shows a gradual increase. The Impella support level is gradually reduced, but blood pressure remains stable, and both CO and CPO are maintained. On hospital day five, Impella is removed, and thereafter, CO and CPO do not decrease and cardiogenic shock dose not recur. BP: blood pressure; PAP: pulmonary artery pressure; RAP: right atrial pressure; PAWP: pulmonary arterial wedge pressure; LVEDP: left ventricular end-diastolic pressure; CO: cardiac output; CPO: cardiac power output; PAPi: pulmonary artery pulsatility index

Hemodynamic data	Day 1	Day 2	Day 3	Day 4	Day 5	Day 6	Day 7
Impella support level	-	P8	P6	P5	P3	-	-
BP (systole/diastole/mean mmHg)	80/64/69	74/57/62	101/78/86	109/71/84	119/73/88	125/74/91	115/57/76
PAP (systole/diastole/mean mmHg)	-	46/31/37	25/15/20	20/7/10	23/10/14	24/11/10	22/15/11
mean RAP (mmHg)	-	17	11	5	10	6	7
mean PAWP (mmHg)	-	29	-	-	-	-	-
LVEDP measured with Impella (mmHg)	-	33	12	8	10	-	-
CO (L/minute)	-	3.1	3.7	4.2	5.0	4.2	4.4
CPO (W)	-	0.57	0.59	0.85	0.94	0.78	0.76
PAPi	-	0.8	0.9	2.6	1.3	2.2	1.0

**Figure 3:**
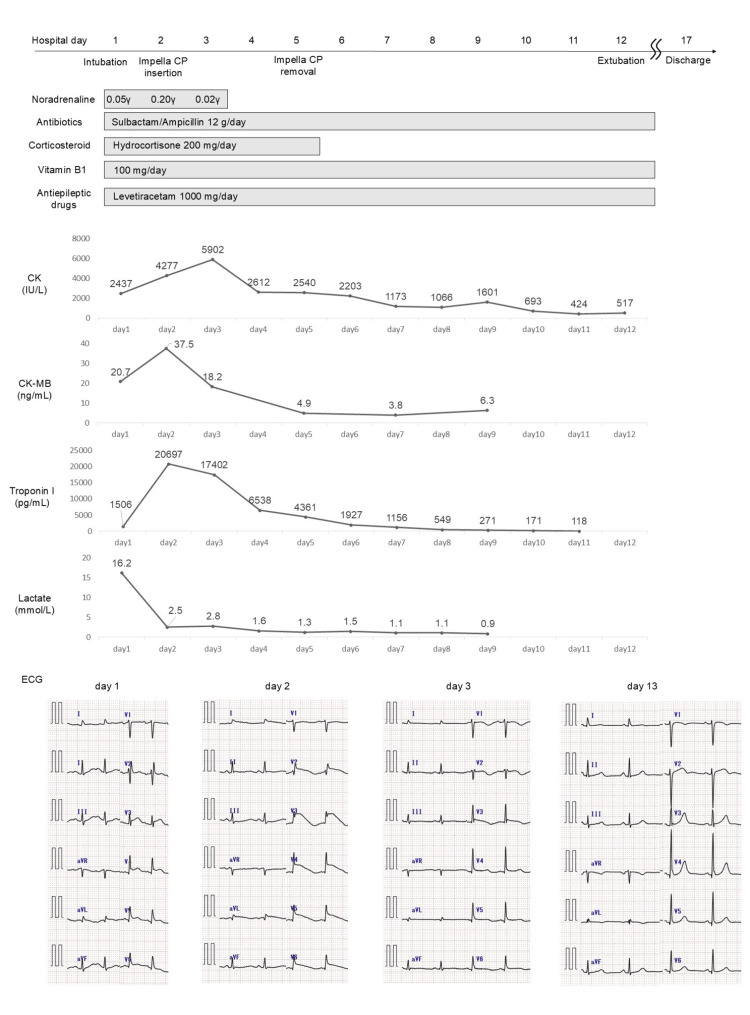
Clinical course after ICU admission. The first row lists major events such as intubation and Impella insertion. The second row provides a list of medications administered. The third row is a graph showing the trends in blood test data. The fourth row shows chronological changes in ECG findings. Immediately after the initiation of mechanical ventilation, the patient begins to show a downward trend in blood pressure. Septic shock due to bacterial pneumonia was suspected, and treatment was initiated with sulbactam/ampicillin, corticosteroids, and norepinephrine. Levetiracetam is started as an antiepileptic, and vitamin B1 supplementation initiated owing to suspected vitamin B1 deficiency resulting from chronic malnutrition associated with excessive alcohol consumption. However, by hospital day two, despite increasing the norepinephrine dose to 0.2 γ, shock persisted. Blood tests reveal elevations in CK, CK-MB, and troponin I, and ECG shows further ST-segment elevation. Based on left ventriculography findings, Takotsubo cardiomyopathy is diagnosed, and an Impella CP device is inserted to provide left ventricular support for cardiogenic shock. By hospital day three, CK-MB and troponin I peaks, and lactate levels are gradually decreasing. Blood pressure also improves, and norepinephrine is discontinued by hospital day three. Extubation is performed on hospital day 12, and on the same day, administration of antibiotics, vitamin B1 supplementation, and levetiracetam discontinued. Thereafter, no recurrence of shock or seizures is observed. Regarding ECG changes, T-wave inversion is noted on hospital day three, and normalized by hospital day 13. ICU: intensive care unit; CK: creatine kinase; CK-MB: creatine kinase-myocardial band; ECG: electrocardiogram

No device-related adverse events were observed, including bleeding or thromboembolic complications. Thereafter, the patient remained hemodynamically stable, with no seizures. The patient was weaned from mechanical ventilation and extubated on day 12 of hospitalization. Transthoracic echocardiography performed on day 13 showed the ejection fraction had recovered to 60%, and the mid-ventricular akinesis had resolved. She was discharged on day 17 (Figure 3).

Electroencephalography performed on day five showed no epileptiform discharges, and brain MRI conducted on day 13 revealed no abnormal signals suggestive of Wernicke encephalopathy. 

## Discussion

Takotsubo cardiomyopathy is a transient myocardial dysfunction that is typically triggered by emotional or physical stress. Several anatomical variants have been reported, with the apical variant being the most common and representative form. In contrast, the mid-ventricular type, as seen in this case, accounts for approximately 10-20% of cases and is more likely to be associated with severe acute left heart failure [7]. There have been reports of an association between alcohol withdrawal and Takotsubo cardiomyopathy. During alcohol withdrawal, enhanced adrenergic activity can lead to excessive catecholamine release, potentially triggering the onset of Takotsubo cardiomyopathy [12]. Additionally, seizures are known to act as triggers because catecholamine levels in the plasma increase during seizure episodes, contributing to the development of this condition [13]. In this case, the patient had a history of abrupt alcohol drinking on the day before the onset of seizures, strongly suggesting a significant contribution of alcohol withdrawal. Although sepsis due to bacterial pneumonia was initially suspected at the time of admission, the subsequent inflammatory response was minimal, blood cultures were negative, and no progression of pneumonia was observed. Therefore, infection-induced Takotsubo cardiomyopathy was considered unlikely. Although the administration of norepinephrine during hospitalization was considered a potential precipitating factor for Takotsubo cardiomyopathy, hypotension suggestive of cardiogenic shock had already developed before the initiation of norepinephrine. Therefore, it was deemed unlikely that norepinephrine was the primary causative factor. Additionally, no significant emotional stressors or other psychological triggers commonly associated with Takotsubo cardiomyopathy were identified during the course. Therefore, Takotsubo cardiomyopathy was considered to have been precipitated by alcohol withdrawal and subsequent status epilepticus caused by acute symptomatic seizures.

A PubMed search using the keywords “takotsubo cardiomyopathy and alcohol withdrawal” revealed only 14 reported cases. The median age was 56 years, with 10 cases involving female patients. In total, 13 cases were classified as apical types, and one case as the focal type. Two patients required mechanical ventilation under intubation; however, none required mechanical circulatory support for cardiogenic shock. All patients recovered, with no fatalities reported. To our knowledge, there have been no prior reports of mid-ventricular Takotsubo cardiomyopathy triggered by alcohol withdrawal and treated with Impella, suggesting that this may be the first such case. Although the overall mortality rate of Takotsubo cardiomyopathy is not high (1.0-4.5%) [1], in certain patients, depending on comorbid conditions or anatomical variants, it may progress to severe heart failure or cardiogenic shock, necessitating mechanical support. Takotsubo cardiomyopathy may involve LVOT obstruction, which can lead to an increased pressure gradient between the left ventricle and aorta, and elevated LVEDP. In such cases, the use of an IABP as mechanical support may worsen the pressure gradient owing to the afterload-reducing effect in systole and potentially result in further hypotension [10,14]. Conversely, the Impella device maintained adequate circulatory flow while unloading the left ventricle. This can reduce the LVEDP without exacerbating the pressure gradient across the LVOT, thereby lowering wall stress and potentially facilitating myocardial recovery [10]. Napp et al. reported the efficacy of Impella support in patients with Takotsubo cardiomyopathy complicated by cardiogenic shock [15]. In the current case, measurement of the aortic-left ventricular pullback pressure revealed both a pressure gradient between the left ventricle and aorta and an elevated LVEDP, suggesting the presence of LVOT obstruction. Moreover, the CO was significantly reduced, and the patient experienced cardiogenic shock. Therefore, mechanical circulatory support was deemed necessary. Given the above findings, IABP was considered unsuitable. The two remaining options were Impella or ECMO. There was no evidence of right ventricular failure or severe respiratory failure; therefore, left ventricular support with Impella alone was sufficient. Considering the less invasive nature of Impella compared to ECMO, an Impella device was selected for circulatory support. Following support, shock resolved, LVEDP decreased, and CO improved, indicating that Impella contributed significantly to the recovery of cardiac function (Figure 3). Moreover, because catecholamine-induced myocardial injury plays a central role in the pathogenesis of Takotsubo cardiomyopathy [1], catecholamines should be used cautiously. In the present case, the use of Impella limited the administration of norepinephrine (Figure 3).

Interestingly, although the blood pressure was maintained upon arrival, arterial blood gas analysis revealed severe lactic acidosis, with a pH of 6.926, HCO₃⁻ of 20.5 mmol/L, and lactate level of 16.19 mmol/L. Chronic excessive alcohol intake promotes the production of reduced nicotinamide adenine dinucleotide (NADH) during ethanol metabolism, leading to an elevated NADH/NAD+ ratio, which impairs the tricarboxylic acid (TCA) cycle and promotes the conversion of pyruvate to lactate. In addition, vitamin B1 (thiamine) deficiency owing to malnutrition can further impair the TCA cycle, resulting in lactic acid accumulation. However, we were unable to confirm thiamine deficiency owing to a lack of pretreatment measurements, so this pathophysiological mechanism remains only a hypothesis. Seizure-induced CO₂ retention and elevated lactate levels may contribute to acidosis. Notably, metabolic acidosis has been suggested to contribute to myocardial injury [16]. In this case, the patient had a mid-ventricular variant of Takotsubo cardiomyopathy, which was more prone to left heart failure. The combination of lactic acidosis due to increased ethanol metabolism and nutritional deficiency (thiamine deficiency) likely exacerbates the left ventricular wall motion abnormalities and contributes to the development of cardiogenic shock. Comprehensive management, including mechanical ventilation, Impella support, vitamin B1 supplementation, and benzodiazepine administration, has enabled successful treatment and survival.

The annual recurrence rate of Takotsubo cardiomyopathy is reported to be approximately 1.0% [17]. The use of angiotensin-converting enzyme inhibitors or angiotensin II receptor blockers has been associated with a reduced risk of recurrence [18]. In the present case, these medications were not administered; however, under psychiatric guidance, she began participating in a self-help group for alcohol dependence and was able to maintain abstinence after discharge. As a result, no recurrence has been observed during the one-year follow-up period.

## Conclusions

Takotsubo cardiomyopathy may be triggered by alcohol withdrawal and subsequent status epilepticus. However, other factors such as infections and medications may also be involved, potentially delaying the diagnosis of Takotsubo cardiomyopathy. If there is an elevation of cardiac enzymes or ST changes on the ECG that do not match the expected clinical course, Takotsubo cardiomyopathy should be considered a differential diagnosis. In addition, depending on the anatomical variant, it can present with left heart failure and cardiogenic shock. Furthermore, lactic acidosis resulting from enhanced ethanol metabolism and vitamin B1 deficiency due to malnutrition may exacerbate cardiogenic shock. In the present case, by maintaining adequate circulatory flow while reducing the LVEDP, the Impella device may have contributed to cardiac recovery and limited the need for catecholamines, making it a suitable form of mechanical support for such pathophysiological conditions. However, given the limited number of reported cases, further accumulation of clinical data is needed to establish its efficacy in similar scenarios. The annual recurrence rate of Takotsubo cardiomyopathy is reported to be around 1.0%, with a mortality rate of 1.0-4.5%. To prevent recurrence, it may be reasonable to consider psychiatric rehabilitation if psychological stress is a contributing factor.
